# Dynamic Psychotherapy: The Therapeutic Process in the Treatment of Obsessive-Compulsive Disorder

**DOI:** 10.3390/bs9120141

**Published:** 2019-12-05

**Authors:** Joana Proença Becker, Rui Paixão, Simone Silva, Manuel João Quartilho, Eda M. Custódio

**Affiliations:** 1 Faculty of Psychology and Education Sciences of the University of Coimbra, 3000-115 Coimbra, Portugal; 2 Psychology Institute of the University of São Paulo, São Paulo 05508-030, Brazil; 3 Faculty of Medicine of the University of Coimbra, 3000-548 Coimbra, Portugal

**Keywords:** dynamic psychotherapy, obsessive-compulsive disorder, treatment, case study

## Abstract

Dynamic Psychotherapy (DP) was developed to overcome the limitations of traditional psychoanalysis, responding to a broader demand of patients who seek help to cope with specific problems in the short term, such as Obsessive-Compulsive Disorder (OCD). OCD is a chronic disabling mental disorder that leads to substantial distress, functional disability and severe occupational and social impairments. Recognizing the literature gap in this field, and the improvements reported by dynamic therapists who have dealt with patients suffering from OCD, a study on the treatment of these patients was conducted in order to discuss the effects of this technique. The method involved a narrative literature review and the analysis of two clinical cases to discuss therapeutic processes, which include the specificities of OCD patients and the mechanisms adopted in the treatment through DP. The therapist’s active stance seemed to be essential to encourage the patient to face feared situations and identify the core conflict. Both patients who were treated through DP presented similarities, such as high anxiety, feelings of guilt and inhibition of aggressive and sexual impulses. Through emotional exploration, confrontation of defensive functioning and interpretative interventions of inner conflicts, patients had reached awareness of their hidden feelings and experiences, and their symptoms and feelings of guilt decreased. They also showed significant improvements in their interpersonal relationships. Although both treatments do not fit into short-term therapies, this technique has led to long-term results, providing evidence that DP may produce favorable outcomes in the treatment of OCD.

## 1. Introduction

Dynamic Psychotherapy (DP) emerged with the objective of overcoming the limitations of traditional psychoanalysis, thereby responding to a broader demand of patients who seek help to cope with specific problems in a short term. Especially when symptoms provoke social and occupational impairments, patients need a short-term solution, making it essential to focus on symptoms and suffering that led them to seek treatment. Dynamic Psychotherapy works in the connection between reality and unconscious fantasies, through a “free interview” and interpretations of transferential reactions and propensities. However, this technique is not only confined to the use of interpretation, but also includes clarification and confrontation as additional therapeutic verbal interventions.

An example of psychopathology in which patients tend to seek for short-term solutions is Obsessive-Compulsive Disorder (OCD), since it leads to substantial distress, functional disability and severe occupational and social impairments [1]. OCD involves psychosocial, genetic and biological factors, in which orderliness, perfectionism, mental and interpersonal control, lack of flexibility and the pursuit for efficiency are the most common features. In addition, the tendency to ruminate and the high need for control over intrusive thoughts and images also integrate with the spectrum of this disorder [2,3,4].

Although there are studies on psychotherapeutic practices in OCD, demonstrating important clinical implications and the reduction of symptoms [5,6,7,8], Dynamic Psychotherapy is not included in first line treatments, possibly due to a lack of studies conducted on this technique. Nevertheless, given this scarcity and considering the improvements reported by dynamic psychotherapists who have treated patients with obsessive-compulsive symptoms [1], research on the treatment of patients diagnosed with OCD was conducted in order to discuss the effects of this technique.

## 2. Methods

This study includes two phases:

### 2.1. The Choice of Theoretical Support

A literature review was conducted in the ProQuest Psychology Journals and Web of Sciences databases with the key words “Dynamic Psychotherapy”, “obsessive-compulsive and psychotherapy” (title), “obsessive-compulsive (title) and psychoanalysis (anywhere)”. The eligibility criteria were (a) original studies on the Dynamic Psychotherapy process, in which therapist interventions and technique features were addressed, and (b) studies on psychotherapies as treatment of OCD in order to verify the specificities described regarding the treatment of patients with this condition. This review considered (c) articles published in the last five years (d) in the languages understood by some of the authors of this paper – English, French, Portuguese and Spanish. Through the review, 118 articles were found. After the exclusion of duplicates, 32 articles met the eligibility criteria, being 23 studies on the technique and effectiveness of Dynamic Psychotherapy (Supplementary Table S1: presents a summary of 21 studies on Dynamic Psychotherapy as 2 studies are divided into 2 published articles.)

It is worth mentioning that this phase had as its main objective the provision of theoretical support for the authors of the current study, who were interested in learning about the most recently published research in this field. In addition, it served to identify the main aspects considered in the descriptions of treatments through Dynamic Psychotherapy, as well as the peculiarities of the patients with OCD, in order to delineate the focus of analysis of the therapeutic processes of this case study.

### 2.2. The Analysis of Two Therapeutic Processes

#### 2.2.1. Selection of the Cases

The eligibility criteria of the clinical cases were patients who met the DSM-5 criteria for diagnosis of OCD, treated through DP for a period between 12 and 24 months, and who presented marked disparities regarding sociodemographic and clinical characteristics. Thus, the cases of an adult woman, divorced and mother of 4 adult children and a teenage boy who lived with his parents were selected for the current study. These dissimilarities allowed processes to be evaluated under different conditions.

The diagnosis of OCD was established through a clinical interview, which inquired about psychiatric history, use of medications and the presence of the following aspects: (a) recurrent and persistent intrusive thoughts, urges, or images, that caused anxiety or distress; (b) attempts to ignore or suppress such thoughts, urges or images; (c) attempts to neutralize intrusive thoughts, urges or images with some action or substitute thought; (d) repetitive behaviors or mental acts performed according to rules, aiming at preventing or reducing anxiety or distress, or preventing some dreaded situation; (e) the identified symptoms were time-consuming or caused impairment in their social and occupational functioning.

All subjects gave their informed consent (patient in case 1, and patient representative in case 2) before they participated in the study. The study was conducted in accordance with the Brazilian Code of Professional Ethics of the Psychologists (Article 16, CFP RESOLUTION No. 010/05), as well as the WMA International Code of Medical Ethics.

#### 2.2.2. Therapists and Therapy

The therapists, who are the authors of this study, have more than 5 years of experience in Dynamic Psychotherapy, and have had the selected cases supervised by therapists with more than 20 years of experience. The treatments consisted of 45 min weekly sessions.

#### 2.2.3. Case Analysis

Case analysis was based on the effects of therapeutic verbal interventions on patients’ thoughts and behaviors, and the improvement of OCD symptoms throughout the therapeutic processes and the six-month follow-up interviews. The patients’ improvement was based on the aspects inquired in the initial clinical interview, i.e., reduction of intrusive thoughts, urges or images, and the attempts (behaviors or substitute thoughts) to ignore, suppress or neutralize them, reduction of anxiety and distress and the improvements in social and occupational functioning.

## 3. Dynamic Psychotherapy

The most recent studies on Dynamic Psychotherapy have pointed out that the premise of this technique is to focus on patients’ needs by conducting psychotherapeutic interventions to a specific goal in an agreement between patient and therapist. This premise aims at providing symptom relief and the resolution of interpersonal conflicts in a shorter time than classical Psychoanalysis or even other dynamic approaches [9]. In the 1970s and 1980s, proposals of Dynamic Psychotherapy models appeared, having intersubjectivity as the main focus, such as the Luborsky’s supportive-expressive therapy and Davanloo’s short-term intensive psychotherapy (ISTDP) [1,10]. The so-called psychodynamic models of change assert that therapist interventions should promote the experiencing and expression of avoided affect [11].

The ISTDP, which is a short-term treatment, performed in less than 40 sessions, is the technique adopted in most of the studies identified in the current review, indicating that it is suitable for the treatment of chronic health conditions and fragile character structure, as well as mixed psychiatric disorders [12,13]. In general, the first session is longer, consisting of an experimental therapy, or trial therapy, in which a psychodiagnostic evaluation can be performed [10,14,15]. According to this method, the path to establishing therapeutic alliance is through overcoming resistances. The main therapeutic interventions, which aim to unlock the unconscious, include confrontation, clarification, challenge to resistance, head on collision, facilitation of emotional experiences and recapitulation [10,15,16,17,18,19,20]. The basis of ISTDP is resistance, transference and unconscious therapeutic alliance [10,19,20,21,22,23], allowing this technique to be considered as an effective psychodynamic therapy, mainly by studies that cover follow-up interviews and post-treatment assessment measures [18,24,25].

The narrative review conducted in this study identified as the main research lines of Dynamic Psychotherapy (Supplementary Table S1) the treatment of Depressive, Anxiety and Personality Disorders, through an average of 20 weekly sessions focusing on unconscious therapeutic alliance for reaching awareness of hidden feelings and experiences. Through emotional exploration, identification of patters in the patients’ life, confrontation of defensive functioning and interpretative interventions about intrapsychic conflicts, the treatments obtained favorable outcomes, with the reduction of symptoms and an increase in patients’ quality of life [11,13,14,16,20,26,27,28,29,30,31].

Regarding the treatment of OCD, the identified studies present a heterogeneity of therapeutic interventions: Buddhist mindfulness practice, pharmacotherapy, individual or group cognitive-behavioral therapy and dynamic psychotherapy [1,5,8,32]. Leichsenring and Steinert (2017, p. 3), who had conducted a study on the treatment of OCD through a short-term DP, approach the psychodynamics of this disorder by highlighting isolation, undoing, reaction formation, rationalization, displacement and regression as further characteristic defense mechanisms. “The superego of patients with OCD was described as rigid and hyperstrict, forbidding aggressive and sexual impulses. In contrast, the ego was characterized as weak, not being able to differentiate between thinking and acting (magical thinking)”. According to these authors, this inability was described by Freud as the ‘omnipotence of thoughts’, a concept later adopted by cognitive models as the so-called thought–action fusion. “Both psychodynamic and cognitive-behavioral models emphasize the role of dysfunctional mental representations of self and others in OCD. However, whereas cognitive-behavioral models focus on conscious experiences, psychodynamic approaches also address unconscious processes” [1] (p. 8).

The studies on Dynamic Psychotherapy emphasize the role of unlocking the unconscious as the path to diverse outcomes including symptom reduction, interpersonal improvements and reduction of psychiatric medications use [16]. This process of unlocking of the unconscious is characterized “as a discrete, observable, in-session event, defined by a specific therapist-patient interaction preceding a major patient communication revealing in depth, affect laden material about past attachment trauma” [31] (p. 101). As stated by Kenny et al. (2014, p. 6), “a person with unconscious, unprocessed emotions from early life does not distinguish the past from the present”, leading to the establishment of relationships based on emotional and defensive templates developed in childhood.

## 4. Clinical Cases

### 4.1. Case 1

When Sara arrived for the first session of therapy, she was 62 years old, she was divorced, a mother of four adult children, and lived with her mother. She had been taking medication for depression and anxiety for the previous 10 years, with pauses defined by her when she felt she did not need it or that the medication did not have the desired effect.

In the first session, the therapist focused on the patient’s symptoms and reason for seeking treatment. Sara said that she had sought psychotherapy due to her feeling very anxious and her main complaint was not being able to go to the beach. She reported feeling anxious just thinking about it and that it was getting worse: “the last vacation I did not even stay there for a weekend”. Sara mentioned that she was very critical and uncomfortable with people’s behaviors. The beach problem would be an attempt of avoiding other family members, seeing as the house belonged to her mother. Throughout the session, Sara explained that she had been in treatment with another psychologist for five years and stopped therapy because “I thought I was better, but the psychologist was not helping, she could not understand my problems anymore”. Later, the therapist discovered that Sara’s psychologist had become pregnant and the pause in the therapy was felt as an abandonment by patient, who stopped the treatment two months after the psychologist’s return. The second and third sessions were marked by Sara’s crying, while describing her past and life choices. She revealed that she did not marry for love, she described sadly that she was afraid to be alone when she was 23 years old and that John offered financial stability. So, she left her old boyfriend and decided to accept the marriage proposal from a man of whom she created false expectations. Sara claimed that she could not even look at her ex-husband because he annoyed her, and she felt bad for having such feelings.

The beginning of the treatment essentially involved anxiety regulation and the establishment of a good therapeutic alliance. Sara did not seem able to initiate a therapeutic process, she was deeply depressed, unable to perceive the dynamic of therapy and respond to the therapist’s questions. The therapist recommended that Sara be followed by a psychiatrist for verification and possible medication adjustment. It took almost two months of weekly sessions for the dynamic psychotherapy to finally begin. The adjustment of medication was important to enable Sara to initiate this process. The focus of Sara’s therapy was on the central conflict behind her need for control, and working through interpretative and confronting interventions of her defensive functioning, mainly the projective identification. Sara, as well as acting with people in her social and family environment, projected on the therapist the feelings she could not deal with, especially the intolerance, which intensified her persecutory state. The therapist, aware of the projected content, in addition to be able to deal with, addressed situations that might arouse such feelings in Sara. Consequently, the therapeutic process served to interpret the communication of Sara’s emotional state, exploring her own feelings through a personality strong enough to contain them (the therapist). In the following sessions, the patient reported feeling a lot of guilt: guilt for having married for the wrong reasons; guilt for not having completed a bachelor’s degree; guilt for never having worked; guilt for her family’s problems. Sara’s body posture and manner of speaking were controlled, and the symptoms she described (anxiety, intrusive thoughts and irritability) seemed to be related to the fear of losing control. The eminent death of her mother, and the possibility of her daughter leaving home disturbed and provoked ambivalent feelings in Sara. In addition to her deep sadness and anxiety, the patient reported that she suffered from the need to constantly organize the house and that she had “some manias”, such as positioning the tablecloth and having a specific order to prepare her breakfast, which she hid from her family, so they would not judge her as “an unreasonable person”.

Sara gradually began to reveal facts about her past in a quest to finding the reasons for her symptoms and suffering. Sara’s childhood was marked by a demand for keeping order, and organizing her toys to avoid disturbing her father. According to Sara, her mother threatened the daughters by saying that her father was coming, and he could punish them. Sara’s father demanded cleanliness and organization and seemed very disturbed when something was out of place. Following her father’s behaviors, when Sara was child, she established an exact place and position for her slippers and other objects in her bedroom. She relates obsessive-compulsive symptoms to genetics, reporting that her sister “is obsessive about cleaning, using bleach in everything and she is always concerned about dust and bacteria in the house”. Sara said that adolescence was even more difficult as she had many allergies on her legs which she tried to hide with socks, and which, in turn, resulted in blood stains. “My parents never cared, but my classmates laughed and looked at me in disgust. At that time, I was crying in secret”.

As an adult, Sara continued keeping her secrets, hiding her suffering of an unhappy marriage, and other consequences from her choices. Sara mentioned that she was very slim, and had a great body when she was young, but in order to relieve anxiety she would eat compulsively, ultimately leading to her to obesity. “When I was compulsive, I could not stop eating. I ate, and ate, and ate. I ate sweets, then salty foods, and then I wanted sweets again. You cannot imagine. Nobody can”. She did, however, feel proud of herself for having lost 30 kg in four months for her daughter’s marriage, just by walking and changing her eating habits. Another aspect that should be highlighted is that Sara had a strong connection with religion, and attended Catholic Mass every Sunday with her mother. She often questioned the attitudes of other believers of her Church and said that the problem of going to church was seeing people she knew. However, when it happened, she tried to leave without greeting, as if she had not seen the person. Although Sara reported feeling a lot of guilt, this feeling was usually replaced by anger and intolerance, especially towards her ex-husband. When reporting the aversion for her ex-husband, she mentioned the sexual life she had to maintain during the marriage, in which she said she had never felt any pleasure.

Throughout therapy, Sara started feeling safe and the setting had become, as she once described, her “confessional”. Recognizing her fear of being abandoned, the therapeutic alliance was built by reinforcing that the therapist was willing to help and pursue her treatment process. Regarding Sara’s fear of losing control, this was addressed as an unreachable goal, showing that unpredictability is part of life and what can be improved is the ability to deal with it. The verbal interventions were used for Sara to confront contradictions in her speech and to raise awareness that her feelings for others could be, in fact, anger and regret for her own choices. When the search for her mother’s acceptance, and the quest for unreachable expectations became the subject of the sessions, Sara’s anxiety increased, and as a result, she presented some episodes of binge eating.

Despite the maintenance of obsessive-compulsive behaviors, in the tenth month of treatment Sara reported feeling less anxiety and, in the thirteen month she was able to have a dialogue with her ex-husband in order to participate in their children’s events. Sara’s relationships improved over the first year of her treatment, and she was becoming able to understand her motivations and reactions in certain situations, which changed her way of facing her feelings. In the subsequent months of treatment, the patient improved social functioning, her obsessive-compulsive symptoms decreased, and the feeling of guilt was replaced by taking responsibility for her choices and their consequences. The treatment lasted 22 months. Information about the six-month follow-up indicated that Sara had maintained her state of improvement.

### 4.2. Case 2

Paul was a 16 year old high school student who lived with his parents when he arrived for the first session of therapy. Paul’s mother had sought public mental health service in light of a set of symptoms presented by her son, such as intrusive thoughts and repeated nightmares associated with religious content. Moreover, excessive isolation and self-examination, a conflicting relationship with his father, low socialization, and a narrow focus on studies, family and his activities in the Church were impairing Paul’s social life. At the time when Paul started his therapy process, he was in treatment with a psychiatrist, who had prescribed a low dose of antidepressant medicine to treat depressive and anxiety symptoms.

In the first sessions, the therapist focused on establishing a therapeutic alliance, while enquiring about the contents of the patient’s thoughts and nightmares. The possibility of talking about his need for control led to an increase in anxiety and feelings of guilt, resulting in many sessions being required until the patient could feel comfortable to confront his feelings and expose his strategies for reaching the dismissal of unpleasant (or sinful) thoughts.

In the following sessions, Paul reported having intense feelings of guilt as a consequence of believing he was unable to meet social and family expectations (“good student, good son”). Since the Children’s Psychosocial Care Center (CAPSi), where the treatment took place, required professionals to conduct interviews with legal representatives of patients under 18 years old, at the pre and post-treatment, two interviews were conducted with Paul’s mother. In one of these interviews, Paul’s mother revealed her desire for Paul to become a Catholic priest, which was in disagreement with the wishes of Paul’s father and Paul himself.

Throughout the treatment, the therapist realized that the inability to meet expectations was mainly related to his mother’s desire, which conflicted with new sensations and sexual feelings characteristic of puberty, when hormonal alteration is responsible for adolescents feeling more intense sexual desire and excitation. The attempt to control and deny his desires and sexual thoughts led Paul to obsessive-compulsive symptoms.

Having been brought up by a mother who praised Catholicism, Paul developed a series of strategies for controlling and disavowing thoughts that might jeopardize the expectation of being a “good son”, such as reading sections of the Bible repeatedly and praying all day long. Furthermore, a disproportionate responsibility impelled Paul to assume excessive concerns for his age. This demand had also become a focus of the psychotherapeutic intervention, in which the analysis of defense mechanisms and transference was considered. Paul presented a rigorous standard of speech about what he believed to be right or wrong, which he had associated with the teachings of the Church since the age of 10. In light of this context, the therapist used clarification and confrontation as the main verbal interventions in order to reduce the rationalization of his thoughts and introduce new perspectives on the interpersonal situations. Recognizing the difficulties imposed by Paul’s environment, mainly beliefs and demands of his mother, the therapist approached social life issues and considered it necessary to suggest the introduction of moments of relaxation and leisure in the patient’s routine.

Progressively, Paul began a rapprochement with his father and included other activities into his routine, such as going to the gym and social events, thereby expanding his core of friends. The encouragement for understanding and elaborating his feelings led the patient to express his thoughts through comic stories. In these stories, the characters, essentially feminine, represented good and evil, as well as erotism and sexuality. Paul’s creativity had provided a way of dealing with his conflicts between religion and sexuality, allowing him to overcome feelings of guilt and shame and to accept the changes his body was undergoing. As a high school finalist, Paul had chosen technology as his professional field. It is important to highlight that Paul’s father was essential in the therapeutic process, and the benefits of their relationship was reflected in the improvement of his other interpersonal relationships. Paul’s psychotherapeutic process lasted 16 months with 45 min sessions. At the end of 1 year of weekly sessions, in light of Paul’s improvements, the therapist proposed the conclusion of the psychotherapeutic process, but Paul claimed that he did not feel confident without the psychotherapeutic support. Thus, the therapy proceeded in the following 4 months in biweekly sessions, spacing out the time until its end, when Paul realized that he was ready to conclude this process.

## 5. Discussion and Conclusions

“Obsessive-compulsive disorder (OCD) is a chronic disabling mental disorder characterized by recurrent obsessions and uncontrolled compulsions such as repetitive behavioral or mental acts performed in response to an obsession” [1] (pp. 1–2). According to Woon et al. (2017), epidemiological studies have pointed that this disorder usually runs a chronic and deteriorating course, causing significant impairment in the quality of life, mainly regarding social functioning, which is corroborated by both cases presented above. The patients Sara and Paul reported problems in social relationships, and both felt the need for isolation and conducted time-consuming rituals that they were compelled to perform as a form of reducing the anxiety associated with their obsessions. In addition, they described their first symptoms in childhood, which became more intense and harmful over the years.

Despite the highly different characteristics of these patients, Sara and Paul share similarities regarding their upbringing, such as the inhibition of their desires and impulses for exploring and expanding their perspectives, the central role of the Catholic religion imposed by a rigorous mother and the unreachable expectations they believed they had to meet. As mentioned by Leichsenring and Steinert (2017, p. 3), quoting Quint (1988), “patients with OCD were inhibited as children in their natural impulses of experimenting, expanding, testing, and trying out, leading to an inability to differentiate between thinking and acting and to the permanent doubt characteristic of OCD”.

Through retelling their stories, mainly regarding their childhood, Sara and Paul were able to face their feelings (sexual desire, anger, fear of being abandoned), and with the therapists’ verbal interventions they could understand the origin of their symptoms, and the function these symptoms may play in their lives. The therapists’ interventions allowed both patients to experience and express their painful avoided affect, to confront contradictions in their speeches and raise awareness that the high expectations they placed on themselves (a willingness to meet their mothers’ expectations) would never be completely fulfilled. The interventions, such as confrontation and clarification, aimed at developing an alternative perspective which was more flexible and adapted to their experiences [27]. However, as stated by Town et al. (2017b, p. 11), “the therapeutic process involves the delivery of specific emotion-focused interventions tailored to a patient’s capacity to process and tolerate feelings”, which is the reason treatments do not have a predetermined duration.

“The normal development in young people, like growth in size, sexual maturity, emotional development, and cognitive capacity, may be potential triggers or amplifiers of psychiatric disorder, or a potential for their subjective quality of life” [29] (p. 2). Sara and Paul grew up with excessive orders and imposition of rules (demands of order, cleanliness and high school performance), making it even more difficult to deal with the challenges of adolescence. Furthermore, they developed under religious values that were reinforced by rigorous mothers. Although Sara was an adult woman, both treatments had moments which focused on relational and internal psychological growth, seeking a way of enhancing patients’ emotional development and helping them to recognize non-healthy patterns of behaviors and thoughts.

Dynamic Psychotherapy has traditionally placed emphasis on insight into relationship patterns and processing avoided emotions. This technique aims at helping patients to enhance self-understanding, gaining insight into their lives and present-day problems, which may allow them not only to maintain therapeutic improvements but to continue to improve after the end of treatment [13,29].

In the clinical cases presented in the current study, longer treatments were required in comparison to most of the therapeutic process indicated by other research in order to achieve changes in patients’ individual and interpersonal functioning, as well as to decrease their symptoms. Thus, both treatments do not fit into short-term therapies since they exceed 40 sessions [12,13]. While Paul needed a few sessions to progressively withdraw from the therapy and therapist, Sara arrived at the treatment with a deep depression that made it impossible to start therapy without adjusting the medication. These aspects show the variety of symptoms and settings that patients with OCD may present and, also, indicate that although Dynamic Psychotherapy may not be a short intervention, it leads to long-term results.

Although clinical experiments have shown that patients with OCD benefit from Dynamic Psychotherapy, this technique is unexplored and thus its effectiveness remains questionable [1,8,31]. In addition to discussing specificities of the technique, this study aims to encourage the development of future research on the effectiveness of DP, especially focusing on the patients’ responses to different verbal interventions. Further research on types and timing of response to the therapeutic interventions may help to outline a suitable treatment manual, perhaps providing a short-term DP treatment for OCD patients.

## Supplementary Materials

The following are available online at https://www.mdpi.com/2076-328X/9/12/141/s1, Table S1: Summary of studies on Dynamic Psychotherapy.
