# Supplementary material for: Dynamic Psychotherapy: The Therapeutic Process in the Treatment of Obsessive-Compulsive Disorder
Source: Behav Sci (Basel). 2019 Dec 5;9(12):141. doi: 10.3390/bs9120141 (PMC6961005; doi:10.3390/bs9120141)
Supplement: Supplementary file 1 [file behavsci-09-00141-s001.pdf]

behavioral sciences

MDPI

Table S1: Summary of studies on Dynamic Psychotherapy.

#	Citation	Design	N	Psychopathology/ Symptomatology	Therapy Design	Diagnostic	Outcomes Assessment	Highlights	Results	Recommendations
1	(Abbas, Kisely, Rasic, & Katzman, 2013)	Process-outcome study	140 patients treated over 9 years	Somatoform disorder; Anxiety disorder; Personality disorder and Major depression	Intensive short-term dynamic psychotherapy (ISTDP), an average of 9.9 treatment sessions.	Clinical interview	Brief Symptom Inventory (BSI) and Inventory of Interpersonal Problems (IIP) were applied at pre and posttreatment. Comparing before to immediately after treatment, the global BSI score went from 1.62 (SD 0.77) to 1.15 (SD 0.81; $p < 0.001$, effect size d = 0.59), whereas the global IIP score went from 1.52 (SD 0.66) to 1.16 (SD 0.76, $p < 0.001$; effect size d = 0.51).	Direct experience of intense mixed feelings toward others; regulating anxiety and blocking defensive avoidance.	This study provides preliminary evidence that trainees can learn a model, that it is effective with their patients early in their career, and that it may lead to cost savings for institutions funding psychiatric training positions.	Non-specified
2	(Abbass, 2016)	Case study	1	Chronic Depression; Medically unexplained symptoms.	Intensive short-term dynamic psychotherapy (ISTDP). 25 sessions	Clinical interview	-	Complex transference feelings, Resistances and Unconscious therapeutic alliance.	Dynamic Psychotherapy is well positioned to activate feelings connected to relational trauma, help overcome complex resistances to attachment, and enable the processing of pathogenic feelings resulting in a new way of relating to oneself and others. Symptomatic relief. In 8-year follow-up after a 25-session treatment	The use of video self and peer review to detail technical intervention and somatic responses is an invaluable instrument to study one's own cases toward optimizing the pace and depth of emotion access and processing in the psychodynamic frame.

									course, patient was vital at age 77, active in charity work and physically robust.	
3	(Abbass, Town, Ogrodniczuk, Joffres, & Lilliengren, 2017)	Naturalistic Observational study	500 patients treated over 9 years	Somatoform disorder; Personality disorder; Major depression; Disorder anxiety generalized	Trial therapy interviews lasting up to 3 h, using intensive short-term dynamic psychotherapy (ISTDP)	Clinical interview based on DSM-IV.	BSI and IPP were used at baseline and at follow-up. Follow-up interviews were typically conducted 1 month after the therapy. Pre and follow-up assessments indicated a symptom reduction. Observed means (SD) baseline and 1-month follow-up for the BSI were 1.63 (0.76) and 1.34 (0.78), respectively, and for the IIP were 1.53 (0.66) and 1.39 (0.73), respectively.	Experiencing and processing the underlying impulses and feelings.	Significant improvements were self-reported on both symptom and interpersonal problem measures. Moreover, the effects seen in the group with major unlocking were moderate to large and occurred in not just a few patients but in one quarter of these tertiary patients.	Future research should include the use of a randomized comparison condition to control for the effects of time passage. It should also further examine predictors of response by operationalizing degree of resistance as a dimensional variable such that the nature of patient resistance and fragility can be more sensitively explored.
4	(Ajilchi, Nejati, Town, Wilson & Abbass, 2016)	Randomized parallel group	32, being 16 with ISTDP and 16 in waitlist	Depressive symptoms and major depression (TDM)	Intensive short-term dynamic psychotherapy (ISTDP), an average of 15 sessions of 60 min.	Clinical interview based on DSM-IV, and the self-report Beck Depression Inventory (BDI-II),	BDI-II, and the computer-based versions of the measures The Wisconsin Card Sorting Task (WCST) and Stroop Task were applied at baseline, posttreatment and at the 12-month follow-up. The results indicated that participants treated with ISTDP had significantly reduced depression severity both at posttreatment (mean = 17.94) and at 12-month follow-up (mean = 18.06). In addition, modest improvements on most tests of executive functioning were identified.	Working through grief about losses and the experience; Processing of buried rage and guilt about rage related to attachment trauma in childhood.	Consistent with previous research in short-term psychodynamic psychotherapy of major depression, the ISTDP treatment arm produced treatment effects greater than controls that are maintained or increase over time. This preliminary result is important given that the treatment is brief and relatively cost effective.	Future studies should endeavor to include assessment of activities of daily living in addition to executive processing. In addition, further research is necessary to examine whether long-term cognitive functional changes are also observed in short-term variants of psychodynamic therapy.

5	(Hersoug, Hoglend, Gabbard, & Lorentzen, 2012)	Randomized parallel group	100 patients — 52 of transference group; 48 of comparison group	Depression; Anxiety; Personality disorder and Interpersonal problems	Dynamic Psychotherapy, an average of 34 weekly sessions of 45 min throughout 1 year.	Structured Clinical Interview for DSM-III-R (SCID-II), and IIP, Global Expectancy Scale (GES), Target Expectancy, Quality of Object Relations Scale, Psychodynamic Functioning Scales (PFS), Working Alliance Inventory (WAI).	Assessments were applied at four times: pre-treatment, posttreatment, 12-month follow-up and 3-year follow-up. 60% obtained clinically significant change in PFS. Regarding IIP, 43% of the patients obtained clinical significant change in IIP. The results about analyses of the impact of the quality of the alliance on treatment outcome 3 years after termination indicated significant associations: (1) PFS: B = 0.7, $F(1, 119) = 3.9$, $p<0.05$; ES = 0.4; and (2) IIP: B = 0.06, $F(1, 97) = 4.4$, $p = 0.04$; ES = 0.4.	Transference group: therapist included himself in interpretive linking of intrapsychic conflicts, direct manifestations of transference, and allusions to the transference. Comparison group: therapist used interpersonal relationships outside of therapy as the basis for similar interventions (extratransference interpretations).	Longer psychodynamic treatment is beneficial especially for patients with Personality Disorders in a long-term perspective.	Further research may investigate whether there is a systematic variation in the impact of Target Expectancy on IIP outcome, depending on high versus low level of Personality Disorder criteria and whether a combination of high scores of Personality Disorder criteria and unrealistic high expectancies may have contributed to the finding.
6	(Hickey, 2015a; 2015b)	Case study	1	Psychoneurotic disorder	Approximately 17 blocks of ISTDP over the span of 3 years. (Each block consists of 5–6 days of intensive, immersion training)	Clinical interview	-	Transference feelings; Unlocking of the unconscious; Treatment of resistance and Unconscious therapeutic alliance.	Without the careful and thoughtful application of MUSC (Multidimensional unconscious structural changes) throughout all phases of the interview, the patient cannot achieve an understanding of their resistances, their destructiveness, and	Non-specified

									their capacity for change in life.	
7	(Hickey, 2015c; 2015d)	Case study	1	Psychoneurotic disorder	Approximately 17 blocks of ISTDP over the span of 3 years. (Each block consists of 5–6 days of intensive, immersion training).	Clinical interview	-	Transference feelings; Unlocking of the unconscious; Treatment of resistance and Unconscious therapeutic alliance	The total removal of resistance in a patient. A patient experienced a primitive stage of rage. The patient had crippling symptom and character disturbances her entire life, but she was fully in touch with these forces and this was very healthy.	More rigorous research is needed into the current techniques and methodologies of ISTDP.
8	(Johansson, Town, & Abbas, 2014)	Process-outcome study	412	Somatoform disorder; Anxiety disorder; Personality disorder and Major depression	Intensive short-term therapy (ISTDP), an average length of treatment of 10, 2 sessions.	Psychodiagnostic non-specified	BSI and IPP were applied at baseline and posttreatment. The results revealed significant within-group effects of ISTDP on both the BSI and the IIP. Pre and posttreatment scores indicated a reduction from 1.60 to 0.92 (mean BSI), whereas the mean IIP score went from 1.51 to 0.99.	Resistance, emotional mobilization and unlocking of the unconscious.	A major unlocking of the unconscious during therapy predicted better treatment outcome.	Further research may establish a causal relationship between outcome and in-session events in ISTDP. Furthermore, standardized ratings of rise in the complex transference feelings and type and degree of resistance should be developed in future research on ISTDP, to enable further knowledge of the mechanisms of change in ISTDP.
9	(Kenny, Arthey, & Abbass, 2014)	Case study	1	Severe performance anxiety	Intensive short-term therapy (ISTDP). 10 sessions, 4 months	Clinical interview	-	Therapeutic focus; active therapist involvement; central dynamic sequence; therapeutic alliance;	As the defenses were overcome and the unconscious feelings were consciously experienced, it was possible working through previously repressed memories. The anxiety and	The authors mentioned that systematic research is required in this area.

								activation of complex transference feelings.	defenses were relinquished, leaving the patient a more integrated and less anxious and defended person.	
10	(Kramer, Pascual-Leone, Despland, & Roten, 2015)	Process-outcome study	32	Adjustment Disorder with depressive mood	Short-term psychodynamic psychotherapy (STPP). One year of weekly sessions.	Structured Clinical interview for DSM-IV.	BDI-II, Symptom Check List-90 (SCL-90), Helping Alliance Questionnaire-II (HAq-II), Classification of Affective Meaning States (CAMS). Using 1 min as the fine-grained unit of analysis, results showed that the experience of fundamentally adaptive grief was more common in the in-session process of patients with good outcome, compared with those with poor outcomes (x^2= 6.56, p = 0.01, d = 1.23). This variable alone predicted 19% of the change in depressive symptoms as measured by the Beck Depression Inventory at the end of treatment.	Emotional exploration and completion; therapeutic alliance; experience of grief; repetitive interpretation of patterns in patient's life.	A patient's grieving experience during the good alliance sessions as a process that leads to symptom change. Even in a good alliance session, the exact timing to facilitate adaptive grief is important.	Further studies should take into account the therapist responsiveness.
11	(Leichsenring & Steinert, 2017)	Exploratory study	-	Obsessive-compulsive Disorder	STPP for OCD based on SE therapy, 24 sessions	Clinical interview is recommended.	-	Therapeutic alliance; core conflict underlying the OCD symptoms; defenses; avoidance components; modification of	Considering the effectiveness of STPP for anxiety disorders, the authors developed a method of STPP specifically tailored to the treatment of OCD, based on SE therapy, and complemented by disorder-specific interventions.	An empirical test in an RCT is required. If the treatment turns out to be efficacious, it would be of interest to investigate which patients benefit from STPP, CBT, or SSRIs in order to offer patients a treatment from which they will benefit most.

12	(Lilliengren, Johnsson, Town, Kisely, & Abbass, 2016)	Process-outcome study	215	Generalized Anxiety Disorder (GAD)	ISTDP, once a week, not time-limited (average = 8.3 sessions). + Pharmacotherapy.	Clinical interview based on DSM-IV.	BSI, IIP and Mobilization of unprocessed complex emotions (MUCE) were applied at pre and posttreatment. The results indicated significant, moderate to large, reductions in psychiatric symptoms and interpersonal problems during treatment. Pre and posttreatment scores indicated a reduction from 1.63 to 1.27 (mean BSI), whereas the mean IIP score went from 1.58 to 1.29.	internalized object relations. Emotion-focused treatment; mobilization of unprocessed complex emotions; defensive operations; in-session processing of intense, conflicted emotions related to attachment trauma.	Significant decreases in psychiatric symptoms, interpersonal problems, and healthcare costs. Therapist's level of experience and skill may influence treatment length.	GAD should be investigated in a randomized controlled design, accounting for time passage and other factors. Future process-outcome studies should use repeated measures.
13	(Meystre, Kramer, Despland, & Roten, 2016)	Exploratory study	6	Depressive Disorder	STPP, 12 weekly sessions. + Pharmacotherapy, supportive interventions twice a week by a psychiatrist resident; 2 weekly 30-min encounters with nurses for developing psycho-educational skills.	Cases were selected from a randomized controlled trial investigation. Diagnostic assessment non-specified.	Assimilation of Problematic Experiences Scale (APES) was applied each therapy session in order to assess the assimilation level of the sample. The values ranged from 1.00 to 2.01. The mean APES rating for the good-outcome cases was M = 1.52 (N = 882; SD = 0.73), and for the poor-outcome cases, it was M = 1.29 (N = 617; SD = 0.72). There was no significant difference between the two groups ($\beta = 0.22$, $p = 0.182$).	Assimilation and new understanding of past experiences; interpretative and confronting interventions;	Achieving the insight level was a rare psychotherapeutic event, appearing from time to time. Insight may have a significant impact, leading patients to some reflection and elaboration outside psychotherapy. The progress was characterized by peaks of assimilation (insight events).	Investigating the role played by the psychotherapist in the assimilation process may help differentiate good- and poor-outcome cases.

14	(Moradian, Salehi, Hasani, Arshadi, & Azin, 2017)	Experimental study	5	Sexual Disfunction	ISTDP, 20 weekly sessions of 120 min.	Clinical interview based on DSM-5, and Female Sexual Function Index (FSFI)	FSFI, Sexual Quality of life–Female (SQOL-F), and Subjective evaluation of partner's sexual function (SEOPSF) were applied at baseline, final session, and 8-week follow-up. The mean scores at baseline, final session, and follow-up session for FSFI werw11.0, 28.4 and29.8, respectively, and for SQOL-F were 39, 99 and 104 respectively. 10.04 at the final session. The mean score at first session SQQL-F was 28 and 32.2 at the final session.	Defenses; resistance; anxiety regulation; pressure to experience the impulse; analysis of conflict; central dynamic sequence.	The process of change had a remarkable growth when the first breakthrough to the unconscious. Only ISTDP has been able to result in a change in sexual quality of life.	ISTDP may implement all psychoeducational and common cognitive-behavioral interventions in a framework while facing sexual dysfunctions.
15	(Ness, et al., 2018)	Experimental study	30	Major Depressive Disorder	STPP, 28 weekly sessions. A parallel work with parents. Antidepressant medication in severe cases.	Mini International Neuropsychiatry Interview (M.I.N.I.) and Structured interview for DSM-IV Personality (SIDP-IV).	PFS, Global Assessment of Functioning (GAF), SCL-90, and BDI-II were applied at pre and posttreatment. The mean scores at the pre and posttreatment for GAF were 59.2 and 67.1, respectively; for GSI (from SCL-90) were 1.3 and 0.81; for BDI were 29.2 and 15.1; and for PFS were 60.8 and 66.0, respectively.	Emotional growth, development, and maturation; self-understanding of interpersonal patterns; increase awareness of own affects; interpretation of unconscious conflicts.	Variations in outcome seem to be influenced by patient's characteristics and by the therapist variables and context factors. Changes in mood and affect may have larger effect size in response to treatment	Further investigation including an assessment of change in adolescents' treatment may contribute to this field and improve clinical interventions.
16	(Perry, Bond, & Presniak, 2013)	Naturalistic Observational study	53	Suicidal phenomena; Depressive; Anxiety; and Borderline Personality Disorders	Long-term Dynamic Psychotherapy, once or twice weekly. Average of duration	Guided Clinical Interview (GCI) based on DSM-IV.	At baseline, and every 6 to 12 months interviews were conducted, using the Longitudinal Interval Follow-up Evaluation Adapted for the Study of Personality (LIFE-ASP),	Therapeutic alliance; reactions to treatment; transference; countertransference;	When suicidal patients do not express negative affections in therapy, therapist may need to actively inquire about such affects or address	Replicating these findings in additional naturalistic treatment trials, varying durations, but with follow-up of sufficient length to ascertain long-term

					therapy was 3 years. Most of sample used psychotropic medications.		The Psychosocial Treatment Interview (PTI) and Therapeutic Alliance Analogue Scales (TAAS). Significant reductions in suicidal and self-destructive phenomena with treatment. At PTI Total Therapeutic Alliance scores of 0.55 (representing 55% of the maximum score) or above, Negative Reaction to Treatment became a progressively Process of recovery from suicidal phenomena stronger and significant predictor of improvement.		defenses inhibiting them in order to provide material for exploration the bases of the conflicts. Higher levels of the alliance promote more effective work with the negative affective material.	outcome, including recovery (maybe 5-year follow-up).
17	(Perry, Bond, & Békés, 2017)	Naturalistic Observational study	51, being 16 with BPD	Borderline Personality Disorder	Long-term Dynamic Psychotherapy, once or twice weekly. Average of duration therapy was 3 years. Pharmacotherapy was included.	Guided Clinical Interview (GCI) based on DSM-IV, and LIFE-ASP.	Borderline Personality Disorder Scale (BPD Scale), SCL-90, Hamilton Rating Scales for Depression (HRSD-21), Hamilton Rating Scales for Anxiety (HRSA), Social Adjustment Scale (SAS),Defense Style Questionnaire (DSQ), and GAF. When individuals with pronounced BPD symptoms begin treatment, they often have higher levels of other symptoms and poorer functioning compared with many other nonpsychotic patient groups. BPD psychopathology was unrelated to the rate of change in both depressive	Patient-therapist interaction; therapeutic alliance; defensive functioning.	The rate of change is similar between patients with and without Borderline Personality Disorder, when the first group began to improve with treatment.	Further investigations could be focus on comparison of different treatments on a core of common measures to analyze the changes and time to remission.

18	(Town, Abbass, Stride, & Bernier, 2017)	Randomized parallel group	60	Depressive Disorder / treatment resistant depression	Intensive Short-Term Dynamic Psychotherapy (ISTDP), 20 weekly sessions of 60 min. Medication was allowed when remains stable.	M.I.N.I. and the Structured Clinical interview for DSM-IV Axis II Personality Disorders.	symptoms (HRSD) and global functioning (GAF). Hamilton Depression Rating Scale (HAM-D) and Patient Health Questionnaire (PHQ-9) applied at baseline, mid-treatment, and posttreatment. The effect of time was significant and negative (HAM-D: B = −3.876, $p < 0.05$; PHQ-9: B = −3.258, $p < 0.05$) indicating that the incidence of depressive symptoms decreased over time. The sample percentages achieving full and partial remission using the HAM-D or PHQ-9-based diagnostic thresholds varied widely by group (e.g., HAM-D full remission, ISTDP 36% vs. treatment as usual (TAU) 3.7%; HAM-D partial remission, ISTDP 48.0% vs. TAU 18.5%; PHQ-9 full remission, ISTDP 32.0% vs. TAU 4.3%; PHQ-9 partial remission, ISTDP 60.0% vs. TAU 8.7%). When modelling these diagnostic outcomes, we found significant interactions between treatment group and time-point, with the odds of partial and full remission diagnoses showing a significantly	Emotional exploration; anxiety tolerance; emotional avoidant behaviors; awareness and capacity to experience emotions that adversely affect mood.	Comparing to TAU (treatment as usual) significantly more patients receiving ISTDP stopped or reduced medications during treatment. The interventions focused on emotional experience seem to be better for these cases than interpretative intervention.	Future research needs to better understand if therapy could have been optimized, extended, or medically augmented to facilitate remission.

							greater increase over time for patients treated with ISTDP as opposed to TAU..			
19	(Town, Abbass, & Bernier, 2013)	Naturalistic study	89	Personality Disorder	ISTDP, an average of 14.9 weekly sessions.	Clinical interview based on DSM-IV.	BSI, IIP, BDI, and Beck Anxiety Inventory (BAI) were applied at pre and posttreatment. For each outcome measure, the ANCOVAs revealed a significant effect of the Major Unlocking Group while holding constant baseline symptom severity and Resistance level: the magnitude of Change Scores was greater in patients with a Major Unlocking response. The strength of the relationship between *Unlocking* Group and magnitude of Change Scores revealed that the Group factor accounted for about 10% of the variance in Change Scores for the BSI-53 and the BDI, 13% for the BAI, and 20% for the IIP-64.	Resistance; exploration and interpretation of hidden, anxiety laden feelings; unconscious therapeutic alliance.	The patients who experienced *unlocking of the unconscious* in therapy sessions had greater symptom reduction, improvement of interpersonal functioning and decrease of healthcare use.	Improvements in future research methodologies may include multiple assessments of in-session process and post-session outcome to analyze change variables.
20	(Town, Salvadori, Falkenström, Bradley, & Hardy, 2017)	Exploratory, process-outcome study	4	Major Depressive Disorder	20 weekly sessions of 60 min.	M.I.N.I. (DSM-IV).	Beck Depression Inventory II (BDI-II), Clinical Outcomes in Routine Evaluation-Outcome Measure (CORE-OM) and Inventory of Interpersonal Problems – Short Circumplex Form (IIP-SC) were applied at pre and posttreatment. Pre-treatment scores on the	Emotion-focused interventions; affect experiencing; therapeutic alliance; transference; removal of resistance;	Peak affect experiencing was associated with improvements in self-reported patient distress. Experiencing of the somatic component of feelings contributes to therapeutic changes.	Further studies on short-term psychotherapy should aim to collect session-to-session data on a large number of therapies.

							BDI-II, CORE-OM and IIP-SC measures indicate that all the participants scored within the clinical range during the baseline phase. Two participants (P1 and P2) showed clinical and statistically significant change from the baseline phase to the posttreatment on all the assessment tools, they are referred to as 'recovered'. No significant change was demonstrated in the results for P3 and P4, they are referred to as 'no change'.	challenge to defenses.		
21	(Wolff, 2013)	Case study	4	Depressive Disorder; Anxiety Disorder.	Non-specified	Standard Psychiatric Evaluation based on DSM-IV.	-	Experiencing avoided feelings; emotional regulation;	Through a carefully monitoring the patient-s moment-to-moment experience in the session is possible to learn when is a good time to press the experience of feelings or to identify and challenge to defenses.	It is essential to the integration of medication and ISTDP ensure that they work together – considering whether medication is aiding the psychotherapy process or limiting its effectiveness.
